# An unusual case of abdominal compartment syndrome from a massive faecaloma

**DOI:** 10.1093/jscr/rjac348

**Published:** 2022-08-06

**Authors:** Fred Jui-Ju Chuang, Aaron Er-Wynn Lim, Michelle Louise Cooper, Phillip Townend, David James Parker

**Affiliations:** General Surgical Department, Gold Coast University Hospital, 1 Hospital Blvd, Southport, QLD, Australia; General Surgical Department, Gold Coast University Hospital, 1 Hospital Blvd, Southport, QLD, Australia; General Surgical Department, Gold Coast University Hospital, 1 Hospital Blvd, Southport, QLD, Australia; General Surgical Department, Gold Coast University Hospital, 1 Hospital Blvd, Southport, QLD, Australia; General Surgical Department, Gold Coast University Hospital, 1 Hospital Blvd, Southport, QLD, Australia

## Abstract

Severe constipation is a frequent presentation but progression into a life-threatening acute compartment syndrome (ACS) is few and far between. This case highlights the typical physiological manifestations of ACS and the immediate benefits of correcting these physiological imbalances through the disimpaction of a massive faecaloma. Furthermore, in adult patients with a history of colonic dysmotility, adult Hirschsprung’s disease should be considered.

## INTRODUCTION

Abdominal compartment syndrome (ACS) from a large faecaloma is rare; however, it has been previously documented [1–5]. We present a case of a young adult who presented to a tertiary hospital in Australia. This case describes the physiological corrections attempted and the surgical escalation involved. This case report is reported in line with the SCARE criteria [6].

## THE CASE

**Figure 1:**
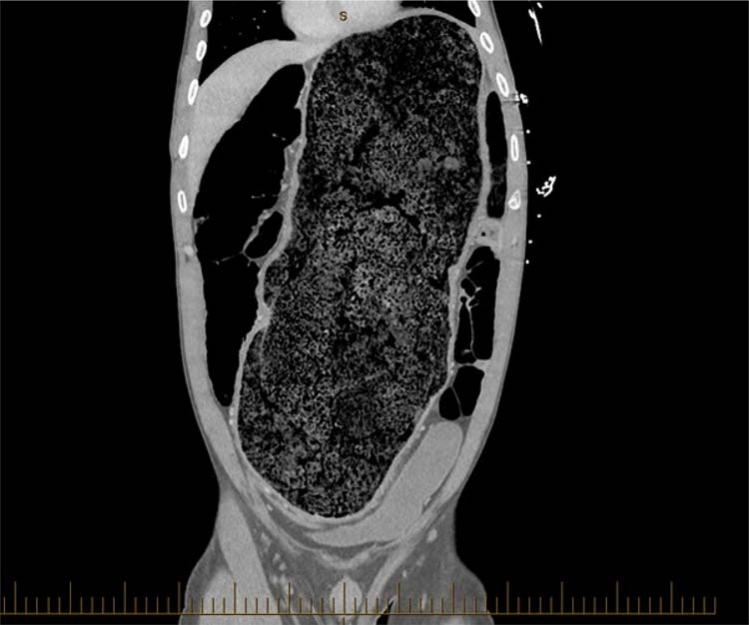
Coronal view of a CT image of a large faecaloma in the sigmoid.

**Figure 2:**
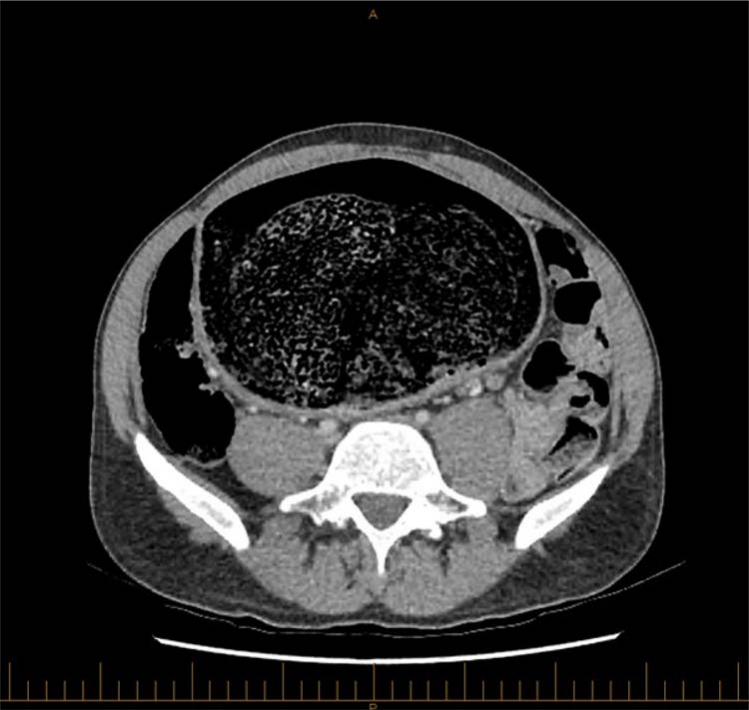
Axial view of CT image of abdomen.

**Figure 3:**
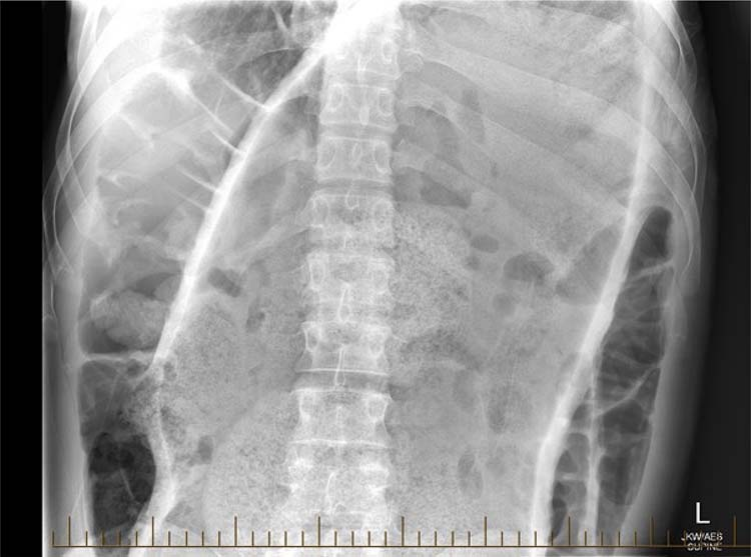
Plain film radiograph of the abdomen.

A 28-year-old gentleman self-presented to the emergency department with profuse overflow diarrhea and vomiting. The patient reported he had normal bowel motion until the morning of presentation. He denied any abdominal pain. On examination, the patient appeared pale, diaphoretic and dehydrated. His abdomen was grossly distended, however, was nontender. He was afebrile yet experienced sustained sinus tachycardia to a rate of 120 beats per minute. His blood pressure sat at 96/75 mmHg and was tachypneic to a respiratory rate of 30 and was saturating at 96% on room air. His blood tests showed a leukocytosis to 31, severe metabolic acidosis pH 7.18, lactate 7.7 and hyperkalemia K 6.0 estimated glomerular filtration rate (eGFR) of 68 mL/min/1.73 m^2^ and a creatinine count of 125μmol/L The computed tomography imaging of his abdomen revealed a massively dilated rectum and sigmoid colon extending to his splenic flexure without evidence of a perforation (Figs 1 and 2). Plain film radiography shows a sizeable faecaloma in the sigmoid colon (Fig. 3).

The patient has a background history of two prior episodes of severe constipation requiring manual disimpaction. He had one attempted colonoscopy in 2017; however, the procedure was aborted due to inadequate bowel preparation; he subsequently failed to attend the follow up colonoscopies.

He suddenly became hypotensive 6 hours postreview in emergency and required the vasopressin, metaraminol support despite 4 L of intravenous fluid resuscitation. His tachypnea progressively worsened and supplemental oxygen was started. The colorectal team was consulted upon, and the decision was made to evacuate the faecaloma in theatre.

Upon manual evacuation of large faecaloma in theatre by the surgical fellow, the patient’s hemodynamics immediately improved and vasopressors were ceased. He was placed on a diet upgrade plan and was placed on an aggressive aperient and enema regime. He was discharged on day 4. He was rebooked for a sigmoidoscopy and biopsy to exclude Hirschsprung’s; unfortunately, he failed to attend the follow-up. On reflection, an opportunistic rectal biopsy during his disimpaction should have been considered.

## DISCUSSION

In all recorded cases of fecal impacted ACS, the diagnosis was made clinically. ACS occurs when intraabdominal pressure exceeds 20 mmHg with end organ dysfunction. Normal physiological abdominal pressures should remain between 0 and 4 mmHg in the adult population [7]. Intraabdominal hypertension causes a series of pathophysiological changes in vascular flow culminating into a perpetuating cycle of increasing pressures and multi end-organ failure. ACS is propagated by increasing venous congestion, third spacing, leading to poor end capillary perfusion which if prolonged may lead to bowel ischemia and perforation [8]. Intraluminal causes of ACS such as that of fecal impaction should be imminently treated with decompression [9]*.*

ACS can manifest vital signs as tachypnea, hypotension and tachycardia. Clinical examination will often reveal a tense abdomen and poor urine output [10]. Expansion of the abdominal cavity causes a cephalad displacement of the diaphragm resulting in a reduction in pulmonary compliance and a reflexive tachypnea. Furthermore, increased pulmonary compression causes a decreased tidal volume and functional residual capacity. Other physiological changes include alveolar atelectasis, increased alveolar dead space, decreased pulmonary blood flow and reduced Co_2_ expulsion. This may lead to a ventilation-perfusion mismatch hypoxemia. Cardiac shock may result from compression of the inferior vena cava resulting in decreased cardiac venous return.

Medical management in ACS can include nasogastric decompression, prokinetic agents, enemas and rectal decompression. The patient’s fluid balance should be closely monitored; and over resuscitation should be avoided to minimize third spacing. In some cases, diuretics may be considered [11].

Measurements of intra-abdominal pressures can be helpful and can be taken either directly with a transducer via an intraperitoneal catheter or indirectly via intravesical transducers.

Adult Hirschsprung disease (AHD) is the most likely etiology for our patient. AHD is a rare gastrointestinal motility disorder characterized by chronic constipation and abdominal distension secondary to the absence of ganglionic cells of the myenteric and submucosal nervous plexus, within segments of bowel. It is commonly identified in neonates and infants, but milder cases can evade diagnosis until acute presentations later in life. The gold standard diagnostic test is a full rectal biopsy but adjunctive tests such as anorectal manometry and a barium enema may be helpful. The common ‘pull-through’ elective surgical management involves removing aganglionic bowel and mobilizing normoganglionic bowel to create an anastomosis above the dentate line.

## CONCLUSION

All general surgeons should familiarize themselves with signs and symptoms of ACS and be able to understand the adjunctive resuscitative measures to manage ACS related physiology. However, prompt manual decompression is often the most important and curative treatment for patients with a large faecaloma.

## CONFLICT OF INTEREST STATEMENT

None declared.

## FUNDING

None.
